# Validation of the Appendicitis Inflammatory Response (AIR) Score in Diagnosing and Classifying Acute Appendicitis: A Prospective Study at a Tertiary Hospital in Bengaluru, India

**DOI:** 10.7759/cureus.109569

**Published:** 2026-05-24

**Authors:** Sudhir Marahanumaiah, Bhoomika Rajkumar, Manoj Mallesh, Nitish Suresh, Kailash Jagannath

**Affiliations:** 1 Department of General Surgery, Kempegowda Institute of Medical Sciences, Bangalore, IND

**Keywords:** acute abdomen, acute appendicitis, appendectomy, appendicitis inflammatory response score, hospitalization, risk assessment

## Abstract

Introduction: Acute appendicitis is a common abdominal emergency that often requires hospital care and urgent surgery. Diagnosis can be difficult, which sometimes results in unnecessary operations. To improve accuracy and reduce avoidable procedures, scoring systems, such as the Appendicitis Inflammatory Response (AIR) score, have been developed, using clinical features and inflammatory markers to guide risk stratification. This study evaluates the AIR score for its ability to distinguish between uncomplicated and complicated cases, rule out non‑appendicitis conditions, and reduce reliance on unnecessary admissions, imaging, and surgery.

Methods: A prospective observational study was conducted at Kempegowda Institute of Medical Sciences and Research Centre, Bengaluru, India, including 100 patients aged ≥ 18 years presenting with right lower quadrant pain, suggestive of appendicitis. Patients were stratified into low, intermediate, and high-risk groups based on the AIR score. Management was tailored accordingly: low scores received outpatient follow‑up, intermediate scores were reassessed with possible imaging or laparoscopy, and high scores underwent immediate surgery. Outcomes included sensitivity, specificity, predictive values, and secondary measures such as negative appendectomy rate.

Results: AIR score ≥ 4 demonstrated sensitivity of 85.9%, specificity of 100%, PPV of 100%, and NPV of 80% (AUC: 0.94) for diagnosing appendicitis. AIR score ≥ 8 showed sensitivity of 85.7%, specificity of 97.6%, positive predictive value (PPV) of 94.7%, and negative predictive value (NPV) of 93.3% (AUC: 0.94) for complicated appendicitis. Negative appendectomy was significantly reduced when the AIR score was applied.

Conclusion: The AIR score is a reliable diagnostic tool for appendicitis, outperforming traditional scoring systems. Its use can reduce unnecessary surgeries, hospital admissions, and imaging, thereby improving patient outcomes and resource utilization.

## Introduction

Appendicitis is increasingly common worldwide, with global prevalence and incidence rising by about 20% since 1990, as per the trends reported by the Global Burden of Disease Study 2019. In 2019, prevalence was 8.7 per 100,000 and incidence 229.9 per 100,000, with Ethiopia, India, and Nigeria showing the largest increases in age‑standardized prevalence [1]. Acute appendicitis remains one of the most common causes of surgical emergencies worldwide and is a leading indication for emergency abdominal surgery, making appendicectomy one of the most frequently performed operations globally [2]. In India, regional studies estimate that 8-12% of emergency department visits are due to acute abdominal conditions, with ureteric colic, appendicitis, and pancreatitis among the most frequently encountered [3]. The identification of appendicitis is complicated by its shared symptoms with various other conditions, especially in the early stages of presentation [4]. Negative explorations (negative appendectomy) occur when surgery is performed for suspected appendicitis, but the appendix is found to be normal. Globally, the incidence has historically ranged between 10% and 20%, though modern imaging and scoring systems, such as the Appendicitis Inflammatory Response (AIR) score, have reduced this to below 5% in many centres. Negative appendectomy is not benign - it exposes patients to avoidable surgical risks, economic costs, and psychological burden. Evidence shows that clinical scoring systems and imaging significantly reduce these harms [5,6]. The negative appendectomy rate for females was 17.64%, while it was 6.06% for males, when surgery was performed based solely on clinical suspicion [7]. This necessitates an accurate diagnosis prior to performing an appendectomy.

Several diagnostic scoring systems have been developed for acute appendicitis to reduce unnecessary appendectomies. Commonly used scores include Alvarado, Modified Alvarado, Lintula, Tzanakis, AIR, Ohmann, Fenyo‑Lindberg, and Raja Isteri Pengiran Anak Saleha Appendicitis (RIPASA), all designed to enhance diagnostic accuracy and lower negative appendectomy rates [8]. The AIR‑S, introduced in 2008 [9], has become the most widely applied pre‑operative tool. Current guidelines from the World Society for Emergency Surgery (WSES) recommend AIR for both diagnosis and management of acute appendicitis [10]. Based on seven clinical and laboratory parameters, patients are stratified into low, intermediate, and high‑risk categories. This study aimed to validate the use of the AIR score in patients with suspicion of acute appendicitis in an Indian tertiary care setting, assessing its diagnostic performance and clinical utility.

## Materials and methods

Study design

This was a prospective observational study conducted over 18 months, from June 2023 to December 2024.

Study site

The study was carried out in the Department of General Surgery, Kempegowda Institute of Medical Sciences and Research Centre, Bangalore, India.

Study population and sample

The study population comprised patients clinically suspected of acute appendicitis. During the study period, 100 consecutive patients meeting the inclusion criteria were enrolled. Adults aged ≥18 years presenting with right lower abdominal pain of less than five days duration were included. Patients with lower abdominal pain lasting more than five days, pregnant women, individuals on immunosuppressive therapy, and those with prior right hemicolectomy or appendectomy were excluded.

Data collection

Data were collected using a pre‑designed semi‑structured proforma. Patient demographics, duration of symptoms, and parameters of the AIR score (Table 1) were documented, including body temperature, neutrophil percentage, C‑reactive protein (CRP) concentration, right lower quadrant pain, rebound tenderness or muscular defense, and history of vomiting.

**Table 1:** Appendicitis Inflammatory Response (AIR) score in patients with suspicion of acute appendicitis WBC: White blood cell; CRP: C-reactive protein

Observation	Criteria	Score
Vomiting	Present	1
Pain in the right iliac fossa	Present	1
Rebound tenderness/guarding	Mild	1
	Moderate	2
	Severe	3
WBC count	10,000-15,000	1
	>15,000	2
Proportion of neutrophils	70-84%	1
	≥85%	2
CRP concentration	10-49 mg/dL	1
	≥50 mg/dL	2

**Figure 1:**
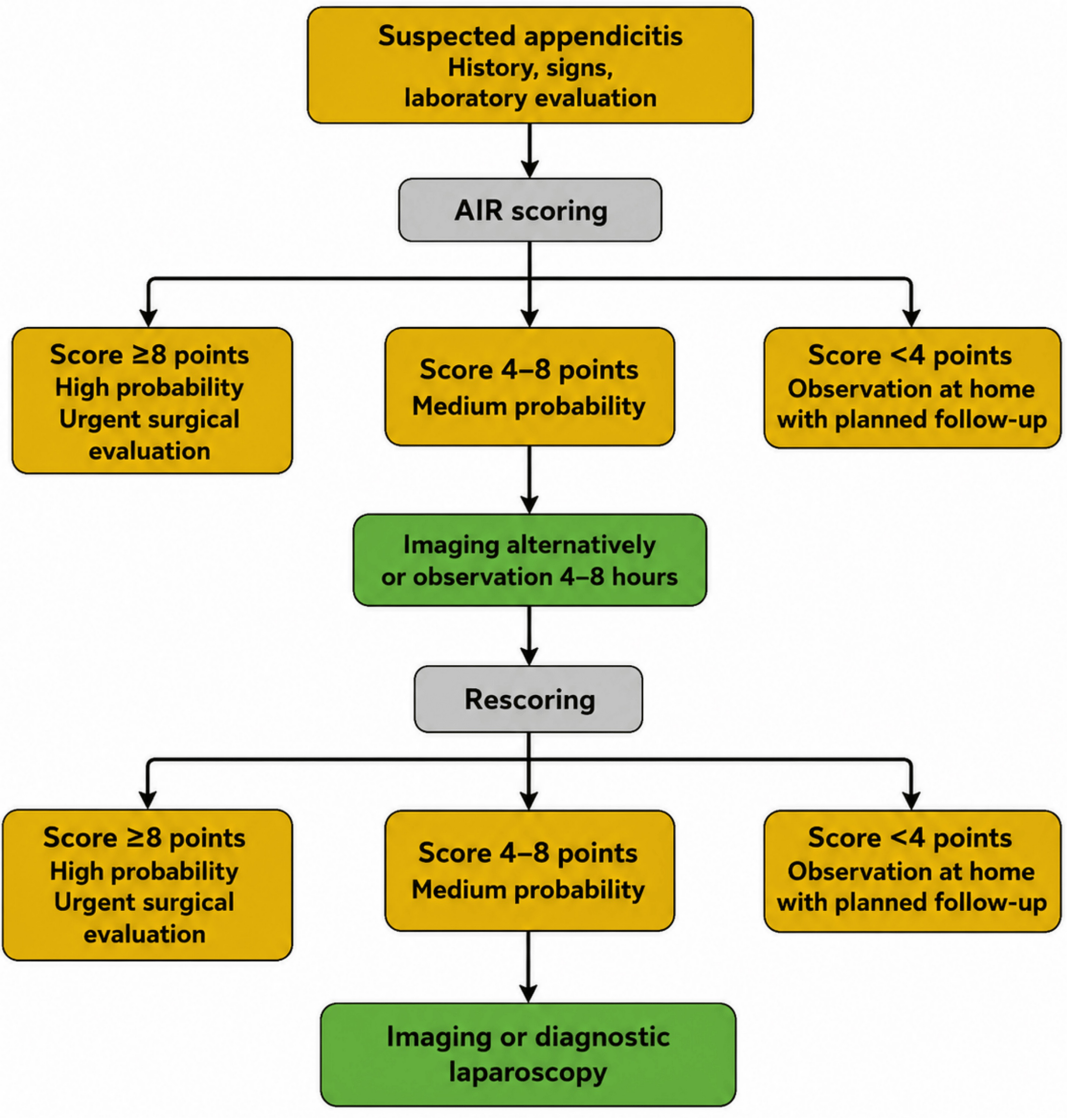
Treatment algorithm with risk stratification based on the AIR score AIR: Appendicitis Inflammatory Response

Figure 1 shows the treatment algorithm with risk stratification based on the AIR score. Patients were stratified into three categories based on the AIR score: (1) low probability (< 5): managed with symptomatic medication, outpatient care, and 24‑hour follow‑up; (2) intermediate probability (5-8): reassessed after four to six hours (further evaluation with imaging or diagnostic laparoscopy as required); and (3) high probability (>8): taken up for immediate surgical intervention.

At discharge, records were maintained on surgical procedures, pre‑ and post‑operative diagnoses, antimicrobial use, and diagnostic imaging (ultrasound and/or computed tomography). All appendices removed were submitted for histopathological examination. The appendix was considered to have burst if a collection of exudate was seen around it or if a perforation with free peritonitis was found after surgery. After imaging revealed an appendiceal mass or abscess, patients with severe appendicitis received drainage and antibiotic treatment.

Histopathological definitions used

Simple appendicitis was defined as transmural neutrophilic infiltration, whereas complicated appendicitis was defined as transmural necrosis or perforation.

Statistical analysis

Clinical, laboratory, and demographic data were analyzed using Statistical Product and Service Solutions (SPSS, version 24.0; IBM SPSS Statistics for Windows, Armonk, NY). Quantitative variables were summarized as mean ± standard deviation, while categorical variables were expressed as absolute numbers and percentages. Associations between categorical variables were examined using the chi‑square test, and comparisons of quantitative data between groups were performed with Student's t‑test. A p‑value of less than 0.05 was considered statistically significant. Diagnostic performance was further assessed by calculating sensitivity, specificity, positive predictive value (PPV), and negative predictive value (NPV). Receiver operating characteristic (ROC) curve analysis was used to evaluate predictive accuracy, with an area under the curve (AUC) of 0.5 indicating no better than chance and values greater than 0.8 reflecting excellent discrimination.

Ethics statement

The study protocol adhered to the principles of the Declaration of Helsinki and was approved by the Institutional Ethics Committee prior to commencement. Written informed consent was obtained from all participants after explaining the study procedures. No additional costs were incurred by participants, and no harm was intended.

## Results

In this study of 100 patients with suspected appendicitis, the mean age was 35.3 years (range: 17-77 years). The majority of patients were aged 21-40 years (n=53, 53%), followed by 41-60 years (n=24, 24%). Males (n=63, 63%) predominated over females (n=37, 37%). Most patients were symptomatic for a duration of two days (n=48, 48%).

All patients (n=100, 100%) had discomfort in the right iliac fossa, persistent tenderness was present in 99 (99%), and vomiting occurred in 75 (75%). Based on the AIR score, patients were categorized as high risk (n=9, 9%), intermediate risk (n=36, 36%), and low risk (n=45, 45%) (Table 2).

**Table 2:** Distribution of patients according to the Appendicitis Inflammatory Response (AIR) score

AIR risk categories	Frequency	Percentage
High	19	19%
Intermediate	36	36%
Low	45	45%
Total	100	100%

Appendicitis was diagnosed in 64 (64%) patients, of whom complicated appendicitis was seen in 21 (33%, Table 3). Among those with alternate diagnoses (n=36, 36%), ureteric calculi (n=13, 36%) and vesicoureteric junction calculi (n=13, 36%) were most common, followed by minor gynecological and gastrointestinal causes; urinary tract pathology was the leading mimic of appendicitis (Table 4).

**Table 3:** Diagnosis of appendicitis vs. complicated appendicitis The Appendicitis Inflammatory Response (AIR) score effectively stratified patients into simple vs. complicated appendicitis. Higher AIR scores (>8) strongly correlated with complicated cases (gangrene/perforation).

Appendicitis	Frequency	Percentage
No	36	36%
Yes	64	64%
Complicated appendicitis	21	33%
Uncomplicated appendicitis	43	67%

**Table 4:** Distribution of patients according to the alternate diagnosis made This distribution highlights that urinary tract pathology is the leading alternate diagnosis in patients presenting with suspected appendicitis. VUJ: Vesicoureteric junction

Alternate diagnosis	Frequency	Percentage
Ureteric calculi	13	36%
VUJ calculi	13	36%
No abnormality	3	8%
Ovarian cyst	2	6%
Ovarian torsion	1	3%
Ureteric stricture	1	3%
Colitis	1	3%
Vesicle calculus	1	3%
Ectopic pregnancy	1	3%
Total	36	100%

Surgical management included laparoscopic appendectomy (n=42, 66%), open appendectomy (n=19, 30%), and conversion from laparoscopic to open (n=3, 5%) (Table 5). Histopathological examination revealed acute appendicitis (n=15, 23%) and periappendicitis (n=17, 27%), which were the most frequent findings (Table 6).

**Table 5:** Surgery performed

Surgery	Frequency	Percentage
Laparoscopic appendectomy	42	66%
Open appendectomy	19	30%
Laparoscopic appendectomy converted to open appendectomy	3	5%
Total	64	100%

**Table 6:** Histopathological report The table shows that most specimens confirmed appendicitis (simple or complicated). Very few cases showed a normal appendix, indicating a low negative appendectomy rate.

Histopathological report	Frequency	Percentage
Acute appendicitis with periappendicitis	17	27%
Acute appendicitis	15	23%
Acute appendicitis with perforation	8	13%
Acute suppurative appendicitis with periappendicitis	7	11%
Subacute appendicitis	6	9%
Gangrenous appendicitis	2	3%
Acute gangrenous appendicitis	1	2%
Acute suppurative appendicitis	1	2%
Acute suppurative appendicitis with abscess	1	2%
Acute suppurative appendicitis with recurrent appendicitis	1	2%
Lymphoid hyperplasia appendix	1	2%
Recurrent appendicitis	1	2%
Recurrent appendicitis with periappendicitis	1	2%
Sloughed off appendix	1	2%
Subacute appendicitis with periappendicitis	1	2%
Total	64	100%

ROC curve analysis demonstrated excellent diagnostic performance. An AIR score ≥ 4 had a sensitivity of 85.9%, a specificity of 100%, a PPV of 100%, and an NPV of 80% (AUC: 0.94) for detecting appendicitis (Table 7). An AIR score ≥ 8 showed a sensitivity of 85.7%, a specificity of 97.6%, a PPV of 94.7%, and an NPV of 93.3% (AUC: 0.94) for complicated appendicitis (Table 8). For predicting the absence of negative appendectomy, an AIR score ≥ 4 exhibited a sensitivity of 94.8%, a specificity of 100%, a PPV of 100%, and an NPV of 66.7% (AUC: 0.98) (Table 9).

**Table 7:** Predictive value of the AIR score for the diagnosis of appendicitis * analyzed using the chi-square test; ** analyzed using the Mann-Whitney test; AIR: Appendicitis Inflammatory Response The table shows that AIR score > 4 predicted appendicitis with high accuracy (AUC: 0.94); all non-appendicitis cases were low risk, showing strong diagnostic correlation.

AIR risk	Appendicitis - No (N)	Appendicitis - Yes (N)	Total (N)	p-value
High	0	19	19	<0.01
Intermediate	0	36	36	<0.01
Low	36	9	45	<0.01
Total	36	64	100	<0.01
Median AIR (mean ± SD)	4 ± 0.6	6.5 ± 2.1	-	-
Cut-off AIR score	≥4			

**Table 8:** Predictive value of the Appendicitis Inflammatory Response (AIR) score for the diagnosis of complicated appendicitis with AIR score (≥ 8) * analyzed using the chi-square test; ** analyzed using the Mann-Whitney test The table shows AIR score > 8 strongly predicted complicated appendicitis; most severe cases were high risk: p < 0.01.

AIR risk	Complicated appendicitis (N)	Uncomplicated appendicitis (N)	Total (N)	p-value
High	18	1	19	<0.001
Intermediate	3	33	36	<0.001
Low	0	9	9	<0.001
Total	21	43	64	<0.001
Median AIR (mean ± SD)	9 ± 1.28	6 ± 1.5	-	-
Cut-off AIR score	≥8			

**Table 9:** Predictive value of the AIR score for the absence of negative appendectomy * analyzed using the chi-square test; ** analyzed using the Mann-Whitney test The table shows that the AIR score > 4 ruled out negative appendectomy, with 94.8% sensitivity and 100% specificity; all false positives occurred in the low-risk group, confirming strong predictive accuracy (AUC: 0.98, p < 0.01). Confirms the AIR score's utility in minimizing unnecessary surgeries.

AIR risk	Negative appendectomy - No (N)	Negative appendectomy - Yes (N)	Total (N)	p-value
High	19	0	19	<0.001
Intermediate	36	0	36	<0.001
Low	3	6	9	<0.001
Total	58	6	64	<0.001
Median AIR (mean ± SD)	7 ± 1.9	4 ± 0.8	-	-
Cut-off AIR score	≥4			

## Discussion

Accurately diagnosing acute appendicitis continues to be a clinical challenge, as errors in diagnosis may result in unnecessary appendectomies or delayed treatment that increases the risk of complications such as perforation and peritonitis [9,10]. This study aimed to validate the AIR score in an Indian tertiary care setting, assessing its diagnostic performance and clinical utility.

In this study, patients were stratified using the AIR score, with 45% classified as low risk, 36% as intermediate risk, and 19% as high risk. Among the 64 patients diagnosed with appendicitis, 64% had confirmed disease, and 33% developed complications. The most frequent alternative diagnoses were ureteric calculi (36%) and vesicoureteric junction calculi (36%), highlighting the AIR score's role in differentiating appendicitis from other causes of right iliac fossa pain.

The present study's AIR score distribution (45% low, 36% intermediate, 19% high risk) closely mirrors findings from other Indian studies, which also reported a predominance of patients in the low-intermediate risk categories with a smaller proportion classified as high risk [11-13]. Among patients without appendicitis, all (100%) were classified as AIR low risk, demonstrating a significant correlation between AIR risk stratification and the final diagnosis of appendicitis. Similar findings were reported by Kabadi et al. and Gupta et al., where low‑risk AIR groups had minimal or no confirmed appendicitis [12,14].

The diagnostic accuracy of the AIR score was high in this study. At a cut‑off of ≥ 4, the AIR score achieved a sensitivity of 85.9%, a specificity of 100%, a PPV of 100%, an NPV of 80%, and an AUC of 0.94, confirming its strong discriminative ability for acute appendicitis. Several Indian studies have reported similar diagnostic accuracy of the AIR score [11-18]: Gupta et al. and Anilkumar et al. found optimal cut‑offs around ≥ 5, yielding sensitivities of ~87% and specificities of ~60% [14,15]; Kabadi et al. noted a sensitivity of 78.6% and a specificity of 85% [12]; and Patil et al. confirmed AIR's superiority over Alvarado and AAS, with an overall accuracy of >90% [16], all consistent with the present study's high sensitivity, specificity, and predictive values.

In this study, none of the patients with complicated appendicitis were AIR low risk, while 86% were high risk and 14% intermediate risk, confirming a strong association between AIR risk categories and final diagnosis. ROC analysis showed that AIR ≥ 8 had a sensitivity of 85.7%, a specificity of 97.6%, a PPV of 94.7%, an NPV of 93.3%, and an AUC of 0.94, highlighting its discriminative accuracy for complicated appendicitis. Similar findings were reported by Meena et al., who observed an AIR accuracy of >90% and superiority over Alvarado and AAS [16], and by de Castro et al., who noted a sensitivity of 89.9% at AIR ≥ 4 and specificity of 100% at AIR ≥ 8 [19], both reinforcing AIR's reliability in identifying complicated cases.

The study findings align with meta‑analyses and systematic reviews, which consistently demonstrate that the AIR score achieves a pooled sensitivity of around 85-90% and a specificity of above 90%, with AUC values exceeding 0.90. These reviews confirm AIR's superiority over other scoring systems, such as the Alvarado, in reducing negative appendectomy rates and guiding risk‑based management [20-23]. The findings however may have limited generalizability as the study was conducted at a single surgical centre, and patient demographics and institutional practices may differ.

## Conclusions

An AIR score of > 4 showed 100% specificity and 85.9% sensitivity for appendicitis, while > 8 predicted complicated appendicitis, with 85.7% sensitivity and 97.6% specificity; thus, AIR scoring is recommended for risk stratification to reduce unnecessary surgeries and enable timely intervention, though multicentre trials are needed to confirm outcome impact.
